# CXCR4 induces cell autophagy and maintains EBV latent infection in EBVaGC

**DOI:** 10.7150/thno.44251

**Published:** 2020-09-18

**Authors:** Weiwen Wang, Yan Zhang, Wen Liu, Xiangyan Zhang, Hua Xiao, Menghe Zhao, Bing Luo

**Affiliations:** 1 Department of Pathogeny Biology, Basic Medicine College,Qingdao University, Qingdao 266071, China.; 2 Department of Clinical Laboratory, Zibo Central Hospital, ZiBo 255000, China.; 3 Department of Pathology, The Affiliated Hospital of Qingdao University, Qingdao 266071, China.

**Keywords:** CXCR4, EBV, EBVaGC, Autophagy, BZLF1

## Abstract

**Rationale:** Epstein-Barr virus (EBV) is found in ~7% of gastric carcinoma cases worldwide, and all tumour cells harbour the clonal EBV genome. EBV can regulate pathways and protein expression to induce gastric carcinoma; however, the molecular mechanism underlying EBV-associated gastric carcinoma (EBVaGC) remains elusive.

**Methods:** GEO microarray and molecular experiments were performed to compare CXCR4 expression between EBV-positive and EBV-negative gastric carcinoma (EBVnGC). Transfections with LMP2A plasmid or siRNA were carried out to assess the role of LMP2A in CXCR4 expression. The effects and mechanisms of CXCR4 on cell autophagy were analysed *in vitro* using molecular biological and cellular approaches. Additionally, we also determined the regulatory role of CXCR4 in latent EBV infection.

**Results:** CXCR4 expression was significantly upregulated in EBVaGC tissues and cell lines. LMP2A could induce AKT phosphorylation to increase NRF1 expression, thereby binding to the CXCR4 promoter to increase its transcriptional level. Moreover, CXCR4 promoted ZEB1 expression to upregulate ATG7 synthesis, which could then activate autophagy. Moreover, CXCR4 increased the number of cells entering the G2/M phase and inhibited cell apoptosis via the autophagy pathway. Finally, CXCR4 knockdown was associated with elevated BZLF1 expression, but this effect was not influenced by autophagy.

**Conclusions:** Our data suggested new roles for CXCR4 in autophagy and EBV replication in EBVaGC, which further promoted cell survival and persistent latent infection. These new findings can lead to further CXCR4-based anticancer therapy.

## Introduction

EBV-associated gastric carcinoma (EBVaGC) accounts for ~7% of all gastric carcinoma cases worldwide 1. Due to the high incidence of gastric cancer, EBVaGC is the most common among EBV-related malignancies 2. The Cancer Genome Atlas (TCGA) divides gastric cancer into four types, namely, EBVaGC, microsatellite unstable tumours, genomically stable tumours, and chromosomally unstable tumours, based on molecular biology 3. Each EBV-carrying gastric carcinoma is of monoclonal origin, arising from a single EBV-infected cell, indicating that EBV may be associated with gastric cancer formation 4. Similar to other herpesviruses, EBV exhibits two modes of infection: latent and lytic infections. The switch from latent to lytic infection starts with the immediate-early gene BZLF1, which activates the expression of EBV immediate-early and early lytic genes 5. In infected tissues, proteins expressed during the lytic and latent viral life cycle lead to cellular alterations that contribute to EBV-associated diseases 6. EBVaGC belongs to the latency I type, with EBNA1 and LMP2A present in 98.1% or 53.8% of cases 7. LMP2A is a transmembrane protein that functions in numerous signal transduction pathways and is involved in EBVaGC tumorigenesis 8. It has been proven that LMP2A induces cell stemness in EBVaGC 9. LMP2A promotes cell survival of B cells through the activation of PI3K/AKT signalling 10. LMP2A was also associated with the induced expression of phosphorylated STAT3 and the upregulation of DNA methyltransferase DNMT1 11 and DNMT3B 12 expression. In summary, LMP2A may play an important role in EBVaGC tumorigenesis.

Dysregulation of genes and pathways plays an essential role in tumorigenesis and tumour progression 13. EBV can regulate host genes and their related pathways to promote cell proliferation and survival, which may directly promote EBVaGC development 14. PD-L1 expression has been observed more frequently in EBVaGC than in EBV-negative gastric carcinoma (EBVnGC) lesions, suggesting that EBVaGC may evade immunological surveillance by T-lymphocytes via the PD-1/PD-L1 pathway 15. Moreover, the canonical NF-κB signalling pathway is constitutively activated, and this plays an essential role in EBVaGC tumorigenesis 16. ADORA1 has been reported to suppress BZLF1 induction, increase tumour cell growth with EBV infection and inhibit sensitivity to the cytotoxic effects of antiviral and anticancer agents 17. Thus, therapy selection based on the mechanism of tumour development is required, and it is important to diagnose EBVaGC prior to treatment.

Recently, it has been reported that EBV latent genes can also modify chemokine receptor expression 18. CXCR4, a seven transmembrane G-protein coupled-receptor, acts as the primary receptor for CXCL12. CXCR4 was first discovered during the HIV epidemic when it was noted that certain strains of the virus used CXCR4 as a co-receptor to infect CD4^+^ T cells 19. It was proven that the peripheral trafficking of CXCR4^+^/CD34^+^/CD133^+^ VSELs was increased in gastric cancer 20 and pancreatic cancer 21, suggesting that CXCR4 may participate in the formation of gastrointestinal cancer. Inhibition of the CXCL12/CXCR4 axis significantly inhibited lymphoma proliferation and survival, suggesting that the CXCR4/CXCL12 axis may participate in EBV-associated lymphomagenesis 22. CXCR4 could modulate the G-protein signalling pathway to influence the invasion and migration of nasopharyngeal carcinoma cells 23. However, the association between CXCR4 and EBVaGC has not yet been reported. Our study aimed to explore the role and molecular mechanism of CXCR4 in EBVaGC tumorigenesis.

## Materials and Methods

### Reagents

Rabbit monoclonal CXCR4 antibody (ab124824), rabbit monoclonal LC3B antibody (ab192890), rabbit monoclonal NRF1 antibody (ab175932), and rabbit monoclonal ZEB1 antibody (ab203829) were purchased from Abcam (UK). Rabbit anti-beta-actin (β-actin) antibody (#4970), rabbit anti-AKT antibody (#4685), and rabbit anti-p-AKT antibody (Ser473, #4060) were purchased from CST (USA). Anti-EBV BZLF1 antibody (sc-53904) was purchased from Santa Cruz Biotechnology (USA). Anti-rabbit and anti-mouse secondary antibodies were purchased from Abcam. Goat anti-rabbit polyclonal secondary antibody conjugated with Alexa Fluor 647 (ab150079) was purchased from Abcam. Anti-rabbit antibody conjugated with Alexa Fluor 555 (#4413) and anti-mouse antibodies conjugated with Alexa Fluor 555 (#4409) were purchased from CST. All antibodies were used according to the manufacturer's instructions. The PI3K/AKT signalling pathway inhibitor LY294002 and EBV reactivation inducer PMA/TPA were purchased from Beyotime Biotechnology (China). Rapamycin (autophagy inducer) was purchased from Apexbio (USA).

### Cell lines and culture conditions

AGS cells containing recombinant neomycin-resistant EBV (AGS-EBV) and control AGS cells were purchased from American Tissue Culture Collection (USA). AGS-EBV cells were classified as latency I type according to the latent products 24. All cell lines were maintained in DMEM (Gibco, Germany) containing 10% foetal bovine serum (Biological Industries, Israel) and 2% penicillin-streptomycin at 37 °C with 5% CO_2_.

### RNA isolation and real-time quantitative RT-PCR

Total RNA was extracted from the cell lines using TRIzol (Invitrogen, USA) and was reverse transcribed using the First Strand cDNA synthesis kit (Takara, Japan). The products were used as templates for the PCR assays using a FastStart DNA Master SYBR Green Kit (Roche, Germany) in the Light Cycler 96 sequence detection system. PCR was carried out following the manufacturer's instructions. All reactions were performed in triplicate, and β-actin was used for normalization. The 2^-ΔΔ*Ct*^ method was used to assess the relative gene expression. The PCR primers are shown in Table 1.

### Western blotting analysis

Protein extraction was performed with a RIPA buffer mixture (RIPA:PMSF:phosphatase inhibitors, 100:1:1), and the proteins were then measured with a bicinchoninic acid assay kit (CWBIO, China). The quantified samples were run on 10% or 12% SDS-PAGE gels and were transferred onto PVDF membranes (Millipore, USA) using a transfer device. The membrane was blocked with 5% non-fat milk for 2 h at room temperature, incubated with the primary antibody at 4 °C overnight and then probed with secondary antibody for 2 h. Proteins of interest were visualized by an enhanced chemiluminescence detection system.

### Gene Expression Omnibus (GEO) microarray dataset analysis

To validate the expression profiles between EBVaGC and EBVnGC tissues, we obtained the GSE51575 microarray from the GEO database (https://www.ncbi.nlm.nih.gov/geo/). We then analysed the mRNA levels of CXCR4 in EBVaGC and EBVnGC tissues.

### Immunohistochemistry

Paraffin-embedded gastric carcinoma tissues were collected from the pathology departments of different hospitals in Shandong Province. EBV-positive cases were identified by *in situ* hybridization for EBV-encoded small RNA1 (EBER1). Sections were incubated for 1 h at room temperature with anti-CXCR4 antibody (1:200). Antigen-antibody complexes were visualized using a DAB chromogenic kit (Zsbio, China). The specimens were randomized, coded, and then analysed by two independent pathologists. The study was conducted in accordance with the ethical standards and the principles of the Declaration of Helsinki and was approved by the Ethical Committee of the Medical College of Qingdao University. Written informed consent was obtained from each participant before the start of the study.

### Plasmid construction

The EBV latent gene LMP2A was cloned into pcDNA3.1 containing the GFP fluorescent protein coding sequence. NRF1 and ZEB1 plasmids were produced by GeneChem Co. (China). Plasmid transfection was performed using the Lipofectamine 2000 transfection reagent (Invitrogen, China) according to the manufacturer's protocols. A total of 2.5 μg of plasmid DNA per six-well plate was used for transfection. Transfection efficiency was measured by qRT-PCR. The cells were harvested after 48 h for downstream analyses.

### RNA interference

The siRNA target sequences for LMP2A and CXCR4 mRNA were designed and synthesized by HANBIO (China). Then, 50 μM siRNA was transfected using Lipofectamine 2000 Reagent. The cells were harvested after 48 h to assay relative gene expression.

### Transmission electron microscopy

Cells were fixed in 2.5% glutaraldehyde at 4 °C, rinsed with PBS, dehydrated in solutions of increasing ethanol concentrations, and embedded in water permeable London Resin White. Samples were sectioned at a thickness of 90 nm, stained with 1% uranyl acetate for 2 min and lead citrate for 5 min and then examined using an electron microscope.

### Immunofluorescence

Cells were grown on cover slips for 12 h, washed with PBS at 37 °C, and fixed with 4% paraformaldehyde at room temperature for 10 min. Fixed cells were blocked in 1% BSA (with 0.15% Triton X-100 and 22.52 mg/ml glycine) for 1 h. The cells were stained with the primary antibodies overnight at 4 °C and incubated with the secondary antibody conjugated with Alexa Fluor 555 for 1 h at room temperature. The nuclei were counterstained with Hoechst 33258 stain (Beyotime Biotechnology, China). Immunofluorescence images were acquired using an Olympus BX63 fluorescence microscope.

### Cell apoptosis detection

For apoptosis detection, 1 × 10^6^ cells with different treatments were seeded into six-well plates, cultured for 48 h, washed twice with PBS, and then detected using the Annexin V-FITC Apoptosis detection kit (BD Biosciences, USA) according to the manufacturer's instructions. After incubating for 10-15 min in the dark at room temperature, specimens were analysed by fluorescence-activated cell sorting (FACS) using a FACSCalibur apparatus (BD Biosciences, USA).

### Propidium iodide staining analysis

Cells were collected 48 h after the indicated treatments, washed with PBS, and fixed in 66% ethanol overnight at 4 °C. Using a propidium iodide (PI) flow cytometry kit (Abcam, USA), the cells were incubated with PI and RNase staining solutions for 30 min at 37 °C in the dark. Finally, based on the standard protocol, > 10,000 cells were detected by FACS using a FACSCalibur apparatus.

### Flow cytometric analysis

For each reaction, 1×10^6^ cells were suspended in 100 μl FACS buffer (1% BSA/PBS). Subsequently, the cells were incubated for 30 min with an anti-CXCR4 antibody or a matching isotype control at 4 °C. After washing three times with FACS buffer, the cells were incubated for 30 min at 4 °C with a secondary goat anti-rat fluorescein isothiocyanate antibody (Alexa Fluor 647) and washed three times with FACS buffer. Cells were detected by FACS using a FACSCalibur apparatus.

### Luciferase reporter assay

To test the effect of transcription factors on the expression of putative target genes, wild-type and mutant 3′-UTR oligonucleotides were subcloned downstream of the firefly luciferase gene in the PGL3-basic vector (GeneChem Co.). HEK293T and AGS cells were seeded into 24-well plates (7 × 10^4^ cells/well), and after 12 h of inoculation, cells were transfected with 1.5 µg of recombinant plasmid (wild-type or mutant 3′UTR), 1.5 µg of NRF1 or ZEB1 plasmid, and 300 ng of pRL-TK plasmid per well. Cell lysates were collected after 48 h, and luciferase activity was detected using the dual luciferase reporter system (Promega, USA).

### Detection of EBV DNA copy number

EBV nucleic acid amplification fluorescence quantification kits were purchased from DaAn (Guangzhou, China). According to the manufacturer's instructions, extracted DNA was added to the corresponding reagent in the kit. Afterwards, the copy number of EBV DNA was determined using the following procedure: 93 °C for 120 s, followed by 10 cycles of 93 °C for 45 s and 55 °C for 60 s and 30 cycles of 93 °C for 30 s and 55 °C for 45 s. The results were calculated according to a standard curve.

### Statistical analysis

Data were analysed using Student's *t*-test and two-way repeated-measure analysis of variance (ANOVA). Analyses were performed using GraphPad Prism software (GraphPad Software, USA). All data are expressed as the mean ± standard error of the mean (SEM).

## Results

### The expression of CXCR4 in EBVaGC tissues and cells

**Figure 1:**
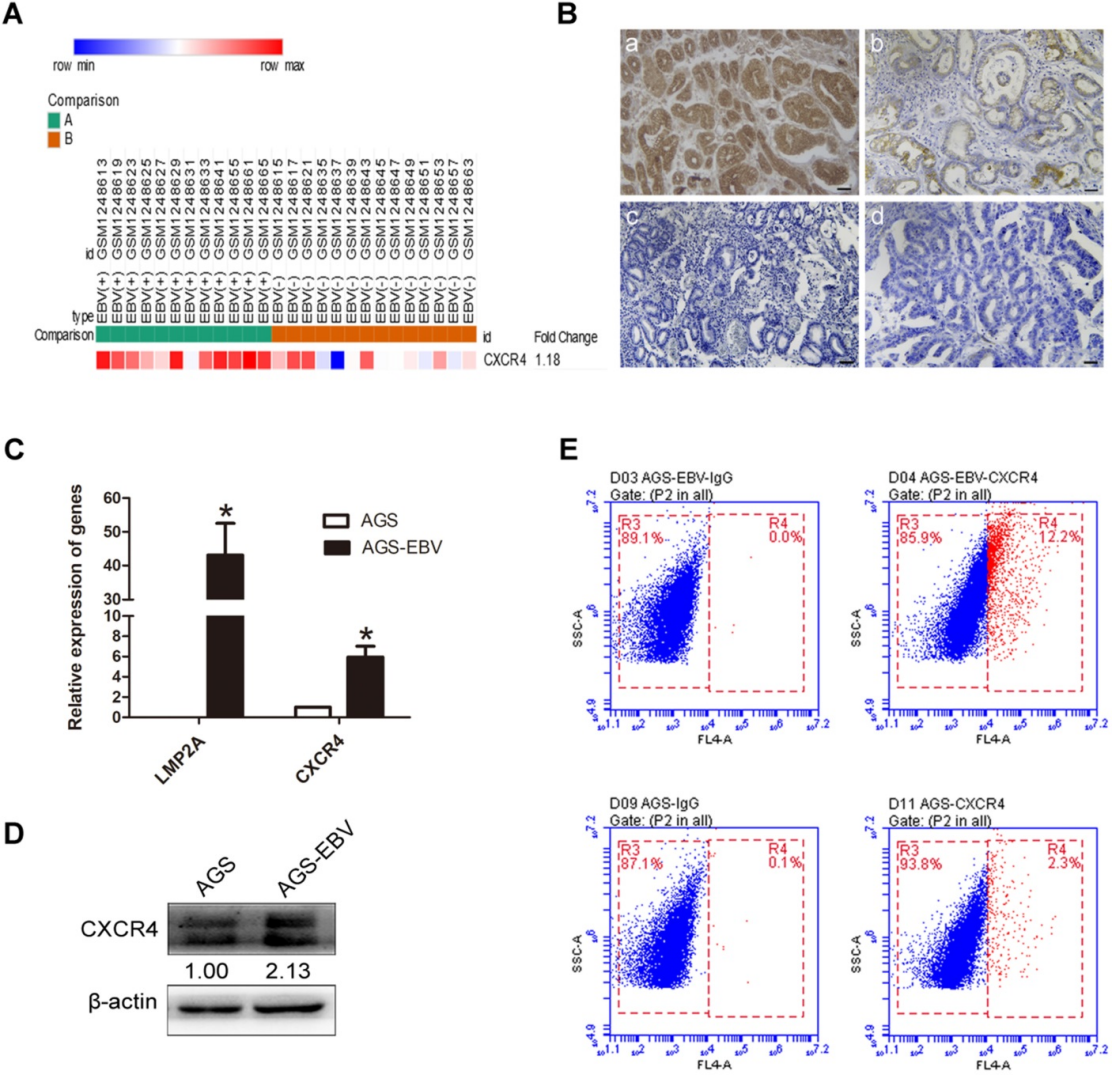
** EBV induces CXCR4 expression in EBVaGC tissues and cells.** (A) CXCR4 expression pattern in GSE51575 (the green group represents EBVaGC tissues, and the red group represents EBVnGC tissues). CXCR4 expression levels decrease from red to blue. (B) The expression of CXCR4 protein between EBVaGC (a, c) and EBVnGC (b, d) tissues determined by IHC. (C) Relative expression levels of LMP2A and CXCR4 mRNA in AGS and AGS-EBV cells. *p<0.05. (D) CXCR4 expression levels were analysed by Western blotting between AGS-EBV and AGS cells. β-Actin was used as a loading control. The average of three independent replicates is shown. (E) The membrane expression of CXCR4 protein was detected using flow cytometry. IgG was used as a negative control.

We downloaded GSE51575 (EBVaGC expression profiling) from the GEO, including 12 EBV^+^ and 14 EBV^-^ gastric carcinoma tissues. The results revealed that CXCR4 was upregulated in EBVaGC compared to EBVnGC samples (Figure 1A). Next, we analysed CXCR4 expression between 38 EBV-positive (proven by *in situ* hybridization with EBER1) and 48 EBV-negative gastric carcinoma patients via immunohistochemistry (IHC), and the results were consistent with the expression found in the GSE51575 dataset (Figure 1B). The expression level of CXCR4 protein is depicted in Table 2. The relationships between CXCR4 expression and patient clinicopathological features, including sex, age, lymph node metastasis, tumour differentiation and invasion depth, were also analysed (Table 3). Finally, CXCR4 expression was significantly higher in AGS-EBV cells than in AGS cells (Figure 1C-D). The surface expression of CXCR4 protein was also detected to verify the above results (Figure 1E).

### EBV increases CXCR4 expression via the PI3K-AKT pathway

**Figure 2:**
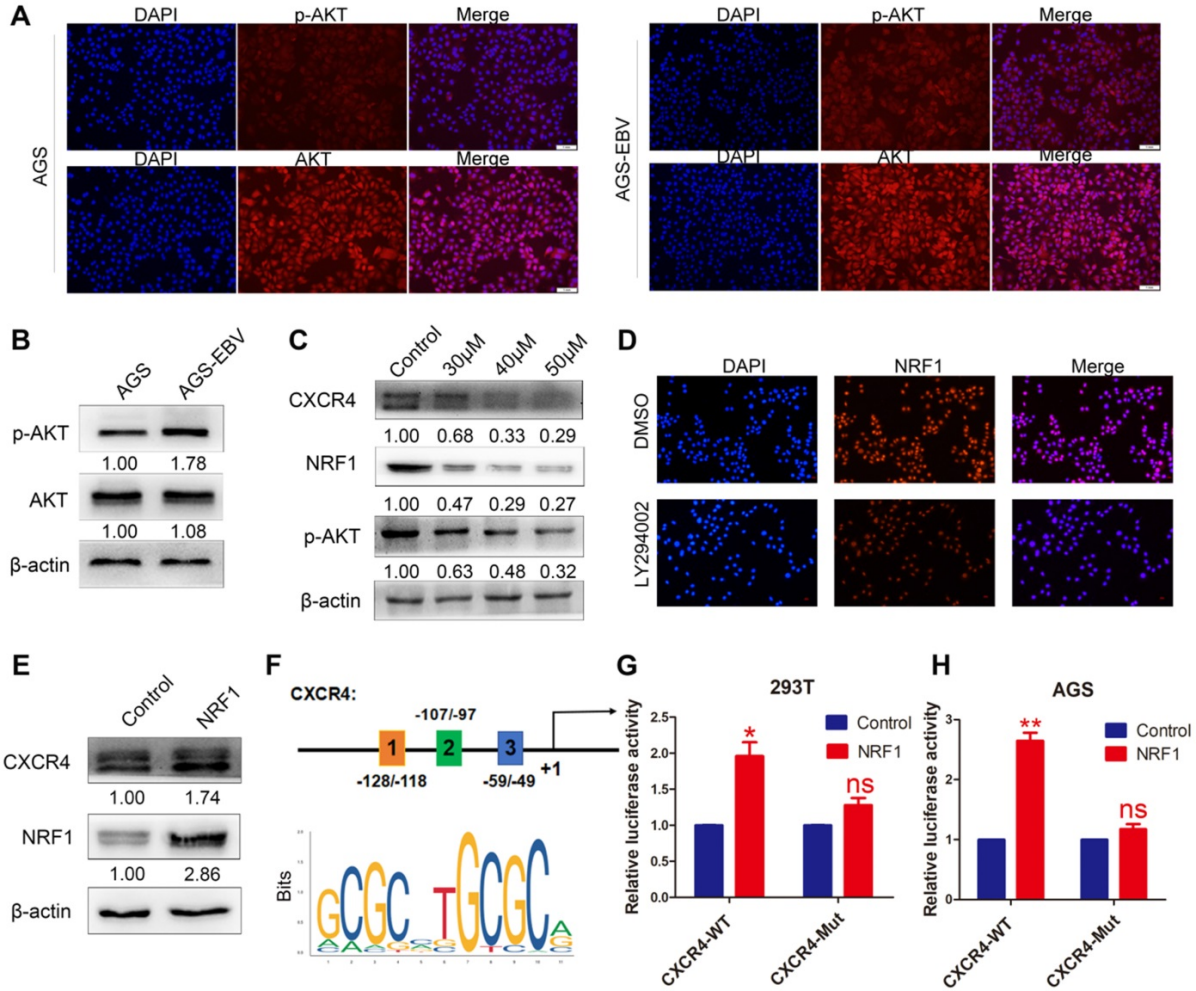
** The effect of PI3K/AKT signalling on CXCR4 expression.** (A) Immunofluorescence staining showed the expression and cellular localization of AKT (red) and p-AKT (red). Cell nuclei were visualized by Hoechst 33258 staining (blue). (B) Western blotting was used to detect AKT and p-AKT expression in EBV-associated GC cells. (C) The EBV-positive cell line AGS-EBV was treated with 30 µM, 40 µM and 50 µM LY294002 for 24 h. CXCR4, NRF1 and p-AKT protein levels were analysed using specific antibodies. Cells were treated with DMSO as a control. (D) Expression and location of NRF1 in AGS-EBV cells treated with LY294002 was detected by fluorescence microscopy. (E) Ectopic expression of NRF1 in AGS cells promoted the expression of CXCR4 protein. (F) Binding sites of NRF1 at the CXCR4 promoter and structure of the CXCR4 promoter luciferase reporter construct. The bottom panel is the consensus binding sequence motif of NRF1. (G) CXCR4 promoter activity was detected in 293T cells overexpressing NRF1 using a dual luciferase reporter system. (H) NRF1 activates CXCR4 promoter activity in AGS cells using a dual luciferase reporter system.

The PI3K/AKT pathway can control a variety of cellular activities, such as proliferation, survival, motility, and morphology. We compared AKT and p-AKT expression between AGS-EBV and AGS cells. As expected, EBV infection led to an increase in AKT phosphorylation (Figure 2A-B). Additionally, we treated AGS-EBV cells with LY294002, which suppressed CXCR4 expression in a dose-dependent manner (Figure 2C), indicating the involvement of PI3K-AKT signalling in EBV-induced CXCR4 upregulation.

To explore the exact role of the PI3K/AKT pathway in CXCR4 expression, we first searched for transcription factor-binding sites (TFBSs) in the CXCR4 promoter using Jaspar (http://jaspar.genereg.net/). PI3K/AKT signalling was confirmed to be necessary for the nuclear translocation of NRF1. Three TFBSs for NRF1 were identified in the CXCR4 promoter (Figure 2F), and the dual luciferase reporter assay showed that upregulated NRF1 expression significantly activated CXCR4 promoter activities in 293T and AGS cells (Figure 2G-H). Additionally, NRF1 expression was suppressed in AGS-EBV cells treated with LY294002 (Figure 2C-D). Additionally, the overexpression of NRF1 could induce CXCR4 expression in AGS cells (Figure 2E). These data suggest that the PI3K/AKT signal is involved in CXCR4 expression via the NRF1 factor.

### The effect of LMP2A on CXCR4 expression

**Figure 3:**
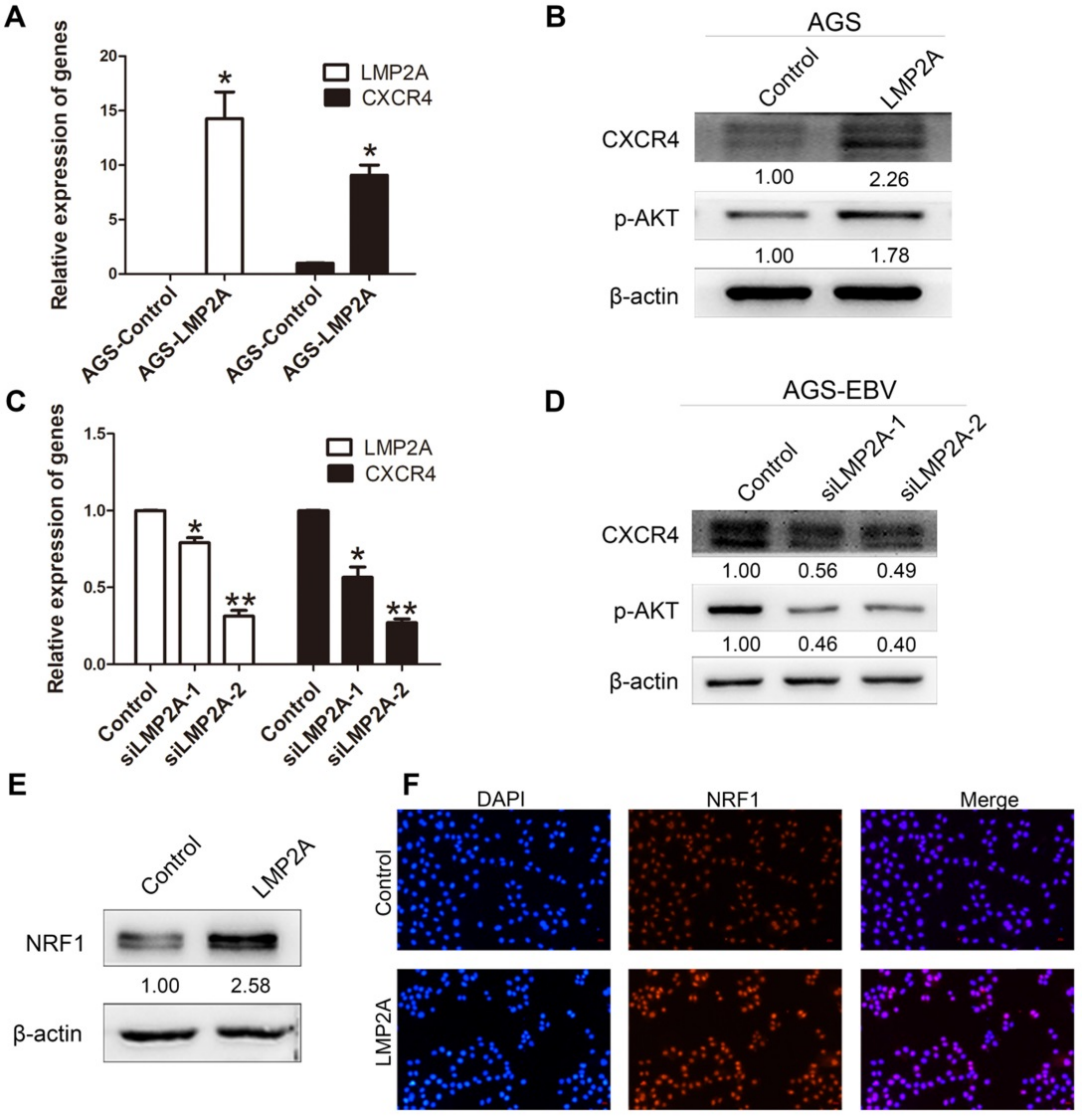
** The role of LMP2A in CXCR4 expression.** (A, B) Detection of CXCR4 and p-AKT expression in AGS cells transfected with LMP2A plasmid. *p<0.05. (C, D) AGS-EBV cells treated with siLMP2A for 48 h were collected to analyse CXCR4 and p-AKT expression. *p<0.05, **p<0.01. The results shown are the averages of triplicate experiments. The mRNA level of LMP2A corresponds to the transfection efficiency. (E) NRF1 expression was detected in AGS cells transfected with the LMP2A plasmid. (F) Expression and localization of NRF1 (red) was detected by Immunofluorescence staining after transfection with the LMP2A plasmid. Cell nuclei were visualized using Hoechst 33258 staining (blue).

To gain mechanistic insights into CXCR4 overexpression caused by EBV infection, we detected CXCR4 and p-AKT expression levels by transfection with LMP2A or siLMP2A. qRT-PCR with primers for LMP2A was used to determine the transfection efficiency. The results showed significant increases in CXCR4 and p-AKT expression in AGS cells following LMP2A plasmid transfection (Figure 3A-B). Moreover, we showed that knockdown of LMP2A (using siLMP2A in AGS-EBV cells) had strong inhibitory effects on CXCR4 and p-AKT expression (Figure 3C-D). Moreover, exogenous expression of LMP2A significantly induced NRF1 expression (Figure 3E-F). Collectively, these results indicated that CXCR4 expression in EBVaGC partly depends on the LMP2A-PI3K/AKT-NRF1 pathway.

### The induction of autophagy by CXCR4

**Figure 4:**
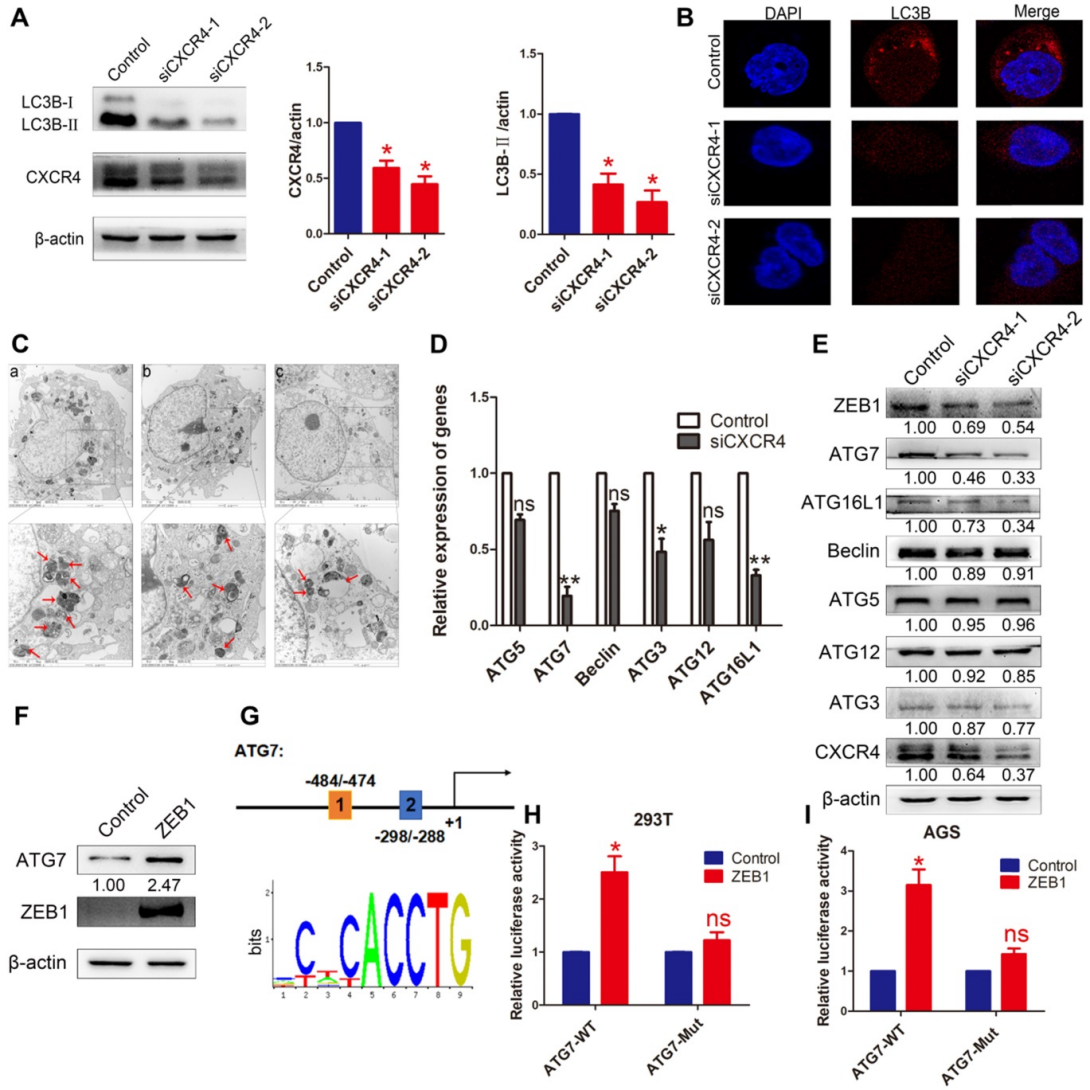
** The effect of CXCR4 on autophagy.** (A) AGS-EBV cells exposed to siCXCR4 for 48 h to detect LC3B-I/II expression. Data are expressed as the mean ± SD. *p<0.05. (B) Immunofluorescence staining was performed to detect autophagy in AGS-EBV cells treated with siCXCR4 and control. (C) Electron micrographs of autophagosome changes in the control (a), siCXCR4-1 (b) and siCXCR4-2 (c) groups. (D) Differential expression of the identified autophagy-related genes was confirmed by qRT-PCR in AGS-EBV cells transfected with siCXCR4 or control. (E) The expression of CXCR4 and its downstream autophagy genes was detected in cells transfected with siCXCR4 and in control cells. (F) Increased ATG7 expression level was observed after ZEB1 overexpression. (G) Structure of the ATG7 promoter reporter plasmid. The bottom panel is the consensus binding sequence motif of ZEB1. (H) Promoter-driven luciferase activity of wild-type and mutant ATG7 in 293T cells. Cells were transfected with ZEB1 or control plasmid. (I) ZEB1 activated ATG7 by binding to the ATG7 promoter, which was detected using a dual luciferase reporter system.

To investigate whether autophagy was induced by CXCR4 in EBVaGC cells, LC3B was detected by Western blotting in AGS-EBV cells transfected with siCXCR4. The assay revealed that siCXCR4 transfection could suppress the autophagy-related protein LC3B (Figure 4A). Furthermore, confocal fluorescence microscopy was used to visualize autophagosome formation in cells treated with control or siCXCR4 (Figure 4B). To confirm these observations, we performed transmission electron microscopy to analyse the samples, as this method is the gold standard for evaluating autophagy (Figure 4C). Compared with those in control cells, the numbers of autophagosomes decreased in the presence of siCXCR4.

To further determine the effect of CXCR4 on cell autophagy, autophagy-related genes were analysed at the mRNA and protein levels after knocking down CXCR4 (Figure 4D-E). The results showed that CXCR4 could significantly induce ATG7 expression. We found ZEB1 binding sites in the ATG7 promoter through Jaspar (http://jaspar.genereg.net/) and constructed the corresponding ATG7 wild-type and mutant reporter plasmids (Figure 4G). The results showed that exogenous expression of ZEB1 significantly promoted the luciferase reporter activity of the ATG7 wild-type promoter construct but not the mutant promoter (Figure 4H-I). We found, for the first time, that the ZEB1 transcription factor activates ATG7 transcription and is involved in autophagy regulation in EBVaGC. Jung et al. previously showed that UHRF1 greatly induced the expression of ZEB1 through CXCR4 25, and our data confirmed these findings. These results indicate that CXCR4 could stimulate ATG7 expression to increase cell autophagy via ZEB1.

### The role of LMP2A in cell autophagy

**Figure 5:**
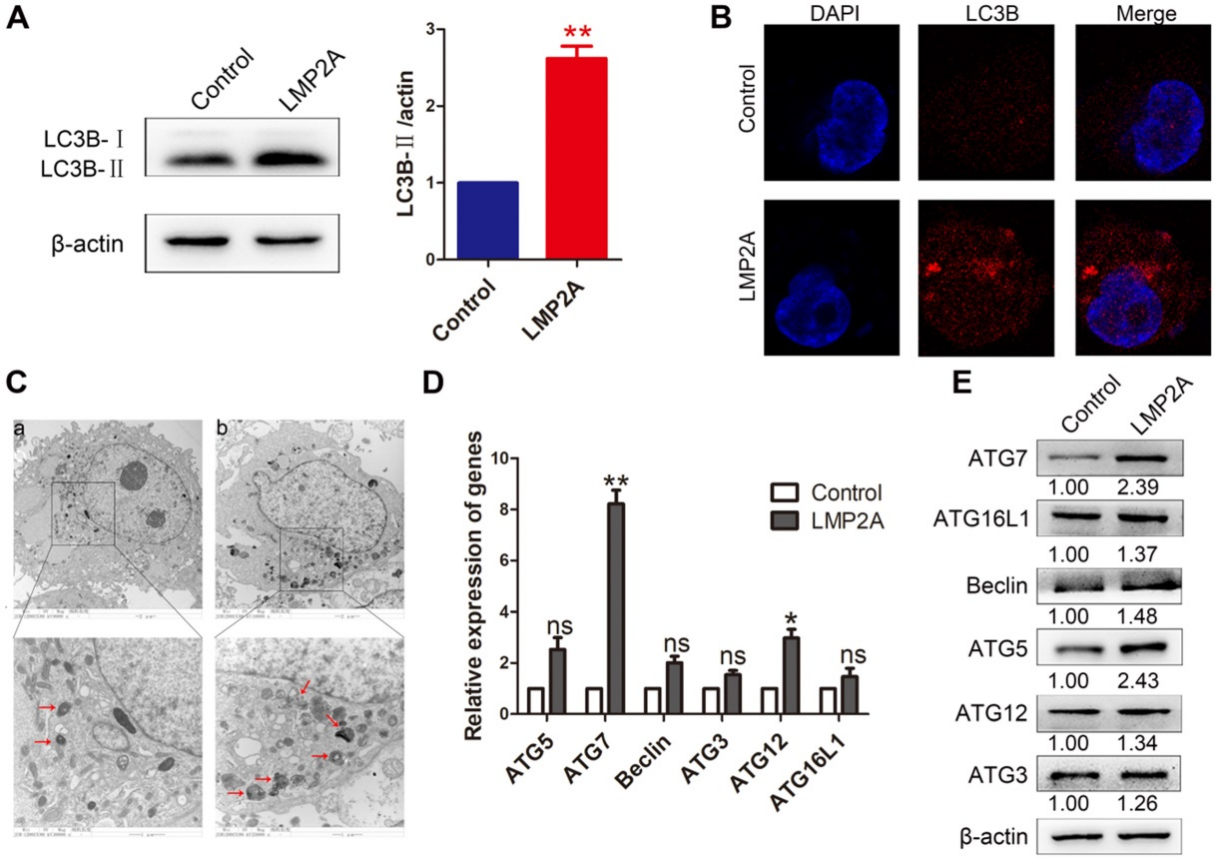
** The effect of LMP2A on cell autophagy.** (A) LC3B-I/II expression was detected in AGS cells treated with LMP2A plasmid. Data are expressed as the mean ± SD. **p<0.01. (B) Immunofluorescence staining was performed to detect autophagy in AGS cells overexpressing LMP2A. (C) Electron micrographs of autophagosomes were analysed in the control (a) and LMP2A (b) groups. (D) The mRNA of the identified autophagy-related genes was confirmed by qRT-PCR in LMP2A-overexpressing cells. (E)Autophagy-related genes were detected in cells transfected with the LMP2A plasmid.

Studies have shown that LMP2A inhibits anoikis and luminal clearing in acini to prevent cell death by inducing autophagy 26. However, the effect of LMP2A on cell autophagy in EBVaGC is still unclear. Our results showed that LMP2A could induce cell autophagy in AGS cells (Figure 5A-C). In addition, LMP2A promoted many autophagy-related genes, especially ATG7 (Figure 5D-E). These data suggest that LMP2A activates autophagy by increasing ATG7 expression.

### The effect of CXCR4 on cell apoptosis and cell cycle progression

**Figure 6:**
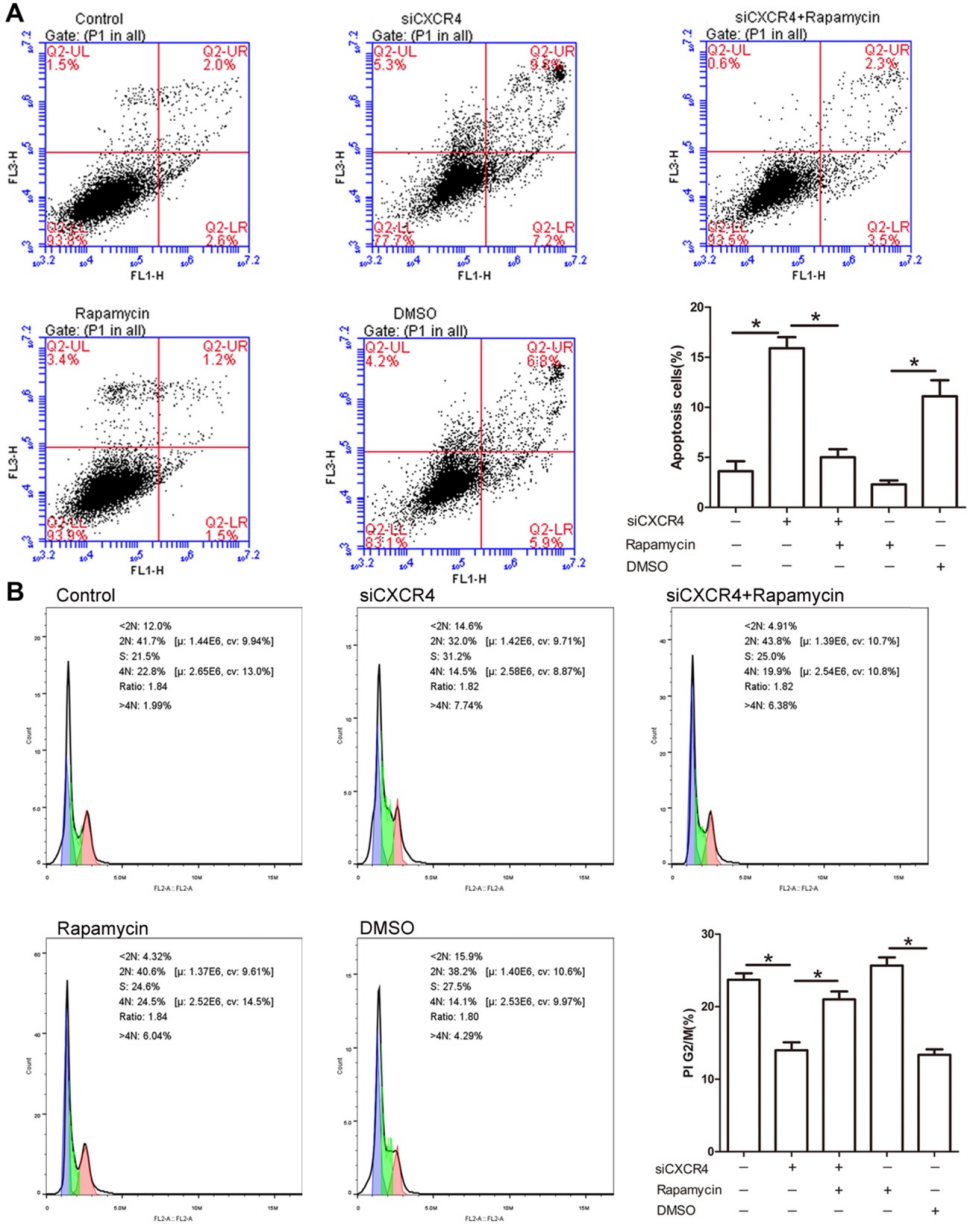
** CXCR4 inhibits cell apoptosis and promotes G2/M arrest.** (A) AGS-EBV cells grown in the presence or absence of siCXCR4 were incubated with either rapamycin or DMSO for the last 6 h of the total incubation time. Apoptosis was measured by Annexin V/PI staining. Representative data are shown as the mean ± SD. (B) PI staining was used to determine the distribution of G2/M cells in the siCXCR4, siCXCR4+rapamycin, rapamycin, DMSO and control groups. Representative data are shown as the mean ± SD.

Due to the potential role of CXCR4 in tumorigenesis, we explored whether CXCR4 affects cell proliferation and apoptosis through autophagy. As depicted in Figure 6A, siCXCR4 treatment resulted in an increase in cell apoptosis, but cell apoptosis was conversely suppressed in siCXCR4+rapamycin (autophagy inducer)-treated cells. Our results suggested that CXCR4 may affect cell apoptosis via autophagy. As shown in the cell cycle assay, transfection with siCXCR4 decreased the accumulation of G2/M cells, and this effect was inhibited by further incubation with rapamycin (Figure 6B). These findings indicated that CXCR4 could also induce G2/M phase arrest by autophagy. The effect of rapamycin on autophagy activation is shown in Figure S1.

### The role of CXCR4 and autophagy in EBV latency

**Figure 7:**
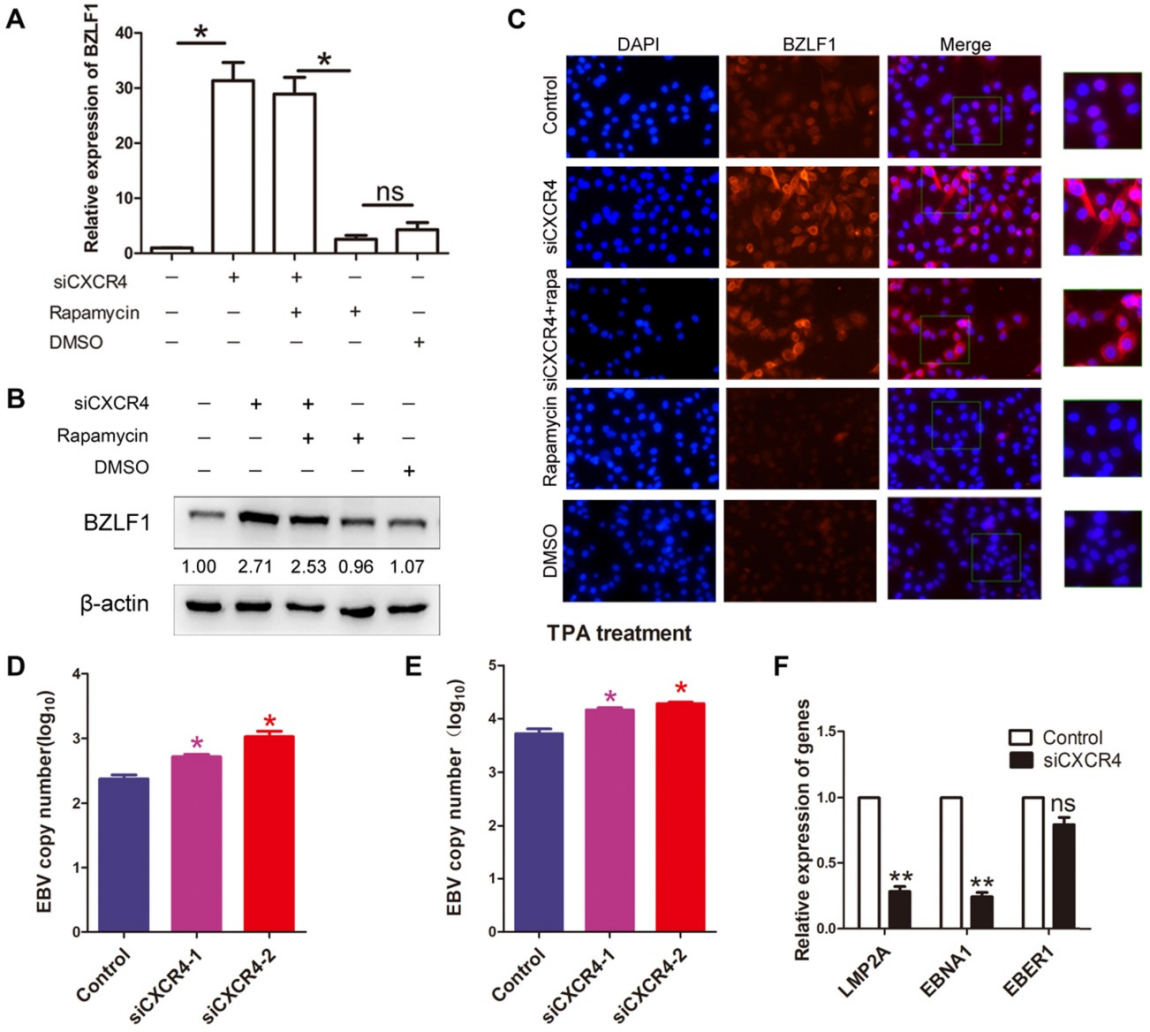
** CXCR4 maintains EBV latent infection.** (A) AGS-EBV cells were treated with siRNA against CXCR4 or negative control siRNA and then incubated with rapamycin for the last 6 h. Quantification of BZLF1 mRNA from three experiments is shown. *p<0.05 (B) Western blotting was used to compare BZLF1 protein expression in the siCXCR4, siCXCR4+rapamycin, rapamycin, DMSO and control groups. The averages of three independent replicates are shown. (C) Immunofluorescence staining was performed to detect BZLF1 (red) expression in AGS-EBV cells treated with five different agents. (D) EBV DNA copy number was determined in cells treated with siCXCR4. (E) EBV DNA copy number was detected by quantitative PCR in siCXCR4 groups when EBV was reactivated by TPA (EBV reactivation inducer). (F) The expression of EBV latent proteins was detected in CXCR4-knockdown AGS-EBV cells.

As it has been hypothesized that autophagy is critical to EBV lytic reactivation and production of viral particles 27; therefore, we wondered whether CXCR4 is involved in EBV latency in EBVaGC via autophagy. AGS-EBV cells were treated with siCXCR4, rapamycin, siCXCR4+rapamycin, or the corresponding controls. A significantly higher BZLF1 expression was observed in cells treated with siCXCR4 than in cells treated with the control. A similar effect was also observed in the siCXCR4+rapamycin group. However, no difference in BZLF1 expression was observed between the rapamycin and DMSO groups (Figure 7A-B). It was further verified through immunofluorescence analysis that BZLF1 expression was upregulated in AGS-EBV cells treated with siCXCR4 and siCXCR4+rapamycin (Figure 7C). The aforementioned data support the notion that rather than autophagy, CXCR4 is required for EBV latency. Additionally, we further examined the effect of CXCR4 expression on EBV latency. Our results showed that CXCR4 could decrease the EBV DNA copy number with or without the presence of TPA (Figure 7D-E). We also detected latency-related genes and found that CXCR4 promoted the EBV-encoded latent genes LMP2A and EBNA1 (Figure 7F). Therefore, CXCR4 may play an important role in maintaining EBV latent infection in EBVaGC development.

## Discussion

We identified new classes of differentially expressed genes (DEGs) through a high-throughput screening of GSE51575, among which CXCR4 was selected for further study. Interestingly, the dataset showed that EBV could induce CXCR4 expression in EBVaGC tissues and cells. The mechanism of EBV and CXCR4 expression has not yet been clearly articulated. Studies have shown that CXCR4 is regulated by many factors. CD164 induces activation of the CXCR4 promoter in the nucleus 28. Extracellular signalling (such as TGF-β) could increase CXCR4 expression in epithelial cells 29. Moreover, EBV-encoded latent products could regulate CXCR4 expression. Nakayama et al. found that stable expression of EBNA2 and LMP1 downregulated CXCR4 expression 30, and it has been proven that transfection of EBNA2 or LMP1 decreased CXCR4 expression in B cell lymphoma-derived cells 31. However, different EBV-related carcinomas show different types of latent infection and express different latent genes. Our study found that LMP2A induced activation of the PI3K/AKT pathway to upregulate NRF1 expression. NRF1 can bind the promoter region of CXCR4 to enhance its transcriptional level. LY294002, an inhibitor of PI3K, effectively reduced NRF1 and CXCR4 expression. Collectively, these findings indicate that LMP2A increased CXCR4 expression via the PI3K/AKT-NRF1 pathway.

Macroautophagy, also called autophagy, is a highly evolutionarily conserved dynamic recycling system for cell homeostasis 32. Autophagy selectively degrades long-lived proteins, removes dysfunctional organelles under basal conditions, and promotes cell survival. Autophagy has been implicated in various human diseases, including neurodegenerative diseases 33, cancers 34, heart failure 35, and inflammatory bowel disease 36. It has been shown that autophagy is dysregulated by EBV to facilitate viral replication and contribute to the onset and maintenance of EBV-associated malignancies 37. We determined that CXCR4 could promote ZEB1 binding to the ATG7 promoter, thereby activating ATG7 transcriptional expression and inducing the formation of autophagosomes in EBVaGC. Fotheringham et al. found that LMP2A also increased autophagosome formation and the expression of proteins in the autophagosome pathway 38. LY294002, an autophagy inhibitor, decreased the numbers of double-membraned autophagosomes and autophagic vacuoles 39. Overall, LMP2A regulation of cell autophagy may partly depend on CXCR4-ZEB1-ATG7 signalling. LMP2A could potentially be required for EBV-induced lymphomas in humans because it is largely dispensable for EBV-induced B cell transformation *in vitro* 40. Due to the survival effect of autophagy, LMP2A takes advantage of the autophagy process to enhance EBV-infected cell immortalization. Previous studies have shown that if autophagy inhibition persists, cells can activate pro-apoptotic genes such as BBC3 via FOXO3 and thus become sensitized to cell death 41. Ravegnini et al. showed that autophagy plays a key role in maintaining gastrointestinal stromal tumour cell survival by providing energy supporting the high energy requirement for metabolism and growth 42. We demonstrated that transfection with siCXCR4 promoted cell apoptosis in AGS-EBV cells, and rapamycin partially inhibited this effect, which revealed that CXCR4 inhibited cell apoptosis by autophagy. Moreover, Zheng et al. reported that there is a clear crosstalk between autophagy and cell cycle regulation, determining cellular life and death decisions 43. Beclin increased several G2/M checkpoint-related proteins, PLK1 and CDC25C, to induce the G2/M cell cycle transition 44. Autophagy induces G2/M cell cycle arrest, accompanied by an upregulation of p21 protein 45. Similarly, CXCR4 induced the number of G2/M cells, and rapamycin enhanced the proportion of G2/M phase cells. These findings indicated that CXCR4 decreased cell apoptosis and increased G2/M arrest in EBVaGC by promoting autophagy.

EBV alternates between latent and lytic modes of infection. During EBV latency, the viral genome is replicated and maintained in circular form at a constant copy number in the absence of virion production 46. EBV establishes latent infection in EBV-associated tumours in the presence of latency proteins 47. BZLF1 expression controls the switch from latent to lytic infection. BZLF1 expression is regulated at the transcriptional level by several activator and repressor proteins that bind various elements of the promoter (Zp). We silenced CXCR4 with siRNA and detected an upregulation of BZLF1 expression, suggesting that CXCR4 acts as a negative regulator of BZLF1 expression. Stephanie et al. showed that C7 reactivated the EBV lytic cycle through subsequent ERK1/2-autophagy activation in EBV-positive epithelial cancers 48. However, De et al. found that inhibition of autophagy enhanced BZLF1 expression and cell replication in EBV-positive Burkitt's lymphoma 49. Based on the mechanism of action in EBV lytic reactivation, lytic inducers can be categorized into autophagy-dependent (C7 and iron chelators) and autophagy-independent (HDACi) subclasses 50. Based on our results, we found that rapamycin has no function in regulating BZLF1 expression, which suggests that siCXCR4 could induce BZLF1 expression by elements other than autophagy. Moreover, inhibition of BZLF1 expression in EBVaGC was beneficial to maintain EBV latency. CXCR4 could decrease the EBV DNA copy number and induce the latent EBV products LMP2A and EBNA1, which confirms the hypothesis that CXCR4 partly contributes to EBV latency.

In conclusion, the key finding in our study is that CXCR4 expression is induced in EBVaGC tissues and cell lines. Moreover, LMP2A promotes CXCR4 transcription via the PI3K/AKT-NRF1 pathway, and CXCR4 activates autophagosome formation by regulating ZEB1-ATG7 transcription. Eventually, CXCR4 inhibits BZLF1 expression and enhances latent gene expression. CXCR4 expression induced by LMP2A could not only contribute to cell growth but also persistently induce EBV latent infection in EBVaGC. Thus, CXCR4 is of importance in EBVaGC development, providing new implications that can be used for novel therapeutic strategies targeting autophagy and EBV reactivation. However, there are still some issues requiring further study. CXCR4 inhibitors or modulators are likely to be toxic, and whether CXCR4 contributes to the prognosis of EBVaGC patients' remains to be elucidated.

## Supplementary Material

Supplementary figure S1.

## Figures and Tables

**Table 1:** List of RT-PCR primers used in the study

PCR Primer	Forward (5'-3')	Reverse (5'-3')
β-actin	TCCTGTGGCATCCACGAAACT	GAAGCATTTGCGGTGGACGAT
CXCR4	GCGTCTCAGTGCCCTTTTGT	TGAAGTAGTGGGCTAAGGGC
BZLF1	CCATACCAGGTGCCTTTTGT	GAGACTGGGAACAGCTGAGG
LMP2A	TGTCGCTGGCATACTCTTCA	GCGTGTTAGTCATCACCGTC
EBER1	AGGACCTACGCTGCCCTAGA	AAAACATGCGGACCACCAGC
EBNA1	GGTCGTGGACGTGGAGAAA	GGTGGAGACCCGGATGATG
ATG3	GTTGGAAACAGATGAGGCTACC	CTCAACTGTTAAAGGCTGCCG
ATG5	ATATCAGACAACGACTGAAAGACCT	TCCAACATTGGCTCAATTCCA
ATG7	GCCAAGATCTCCTACTCCAATC	CAGAAGTAGCAGCCAAGCTTGT
ATG12	TGGAACTCTCTATGAGTGTTTTGG	AGCTGTCTCTTCCGTGAAAATC
ATG16L1	TACGCAGCAAAGTCTGCATAAA	CCAGGGCAGTAATCTTTCCCA
Beclin	AAAACCAACGTCTTTAATGCAAC	GGAACAAGTCGGTATCTCTGAA

**Table 2:** The CXCR4 expression between EBVaGC and EBVnGC tissues

Indicator	EBVaGC (n=38)	EBVnGC (n=48)	*P* value
**CXCR4 expression**			<0.05
3+	10 (26.3%)	0	
2+	7 (18.4%)	11 (22.9%)	
1+	10 (26.3%)	13 (27.1%)	
-	11 (29%)	24 (50%)	

EBVaGC EBV-associated gastric cancer, EBVnGC EBV-negative gastric cancer.

**Table 3:** Association between CXCR4 expression and clinicopathological parameters of EBVaGC and EBVnGC patients

Indicator	CXCR4 expression		*P* value
	Positive (n=51)	Negative (n=35)	
**Gender**			0.313^b^
Male	41	31	
Female	10	4	
**Location**			0.167^b^
Cardiac and fundus	12	4	
Body	15	10	
Antrum	14	16	
Residual and multiple lesions	10	5	
**Age (years)**			0.272
≤60	23	20	
>60	28	15	
**Lymph node metastasis**			0.028
Yes	34	15	
No	17	20	
**Differentiation grade**			0.988^b^
Well and Moderately	6	5	
Poorly	45	30	
**Invasion depth**			0.696^b^
Myometrium	7	3	
Serous layer and above	44	32	

^b^ [missing description].

## Abbreviations

DEGs: Differently expressed genes
EBV: Epstein-Barr virus
EBVaGC: EBV-associated gastric cancer
EBVnGC: EBV-negative gastric carcinoma
EBER1: EBV-encoded small RNA1
GEO: Gene Expression Omnibus
TCGA: The Cancer Genome Atlas
TFBSs: Transcription factor-binding sites
