# Supplementary material for: CXCR4 induces cell autophagy and maintains EBV latent infection in EBVaGC
Source: Theranostics. 2020 Sep 18;10(25):11549–61. doi: 10.7150/thno.44251 (PMC7545993; doi:10.7150/thno.44251)

Figure S1. The effect of different concentration Rapamycin on autophagy activation by Western blotting.

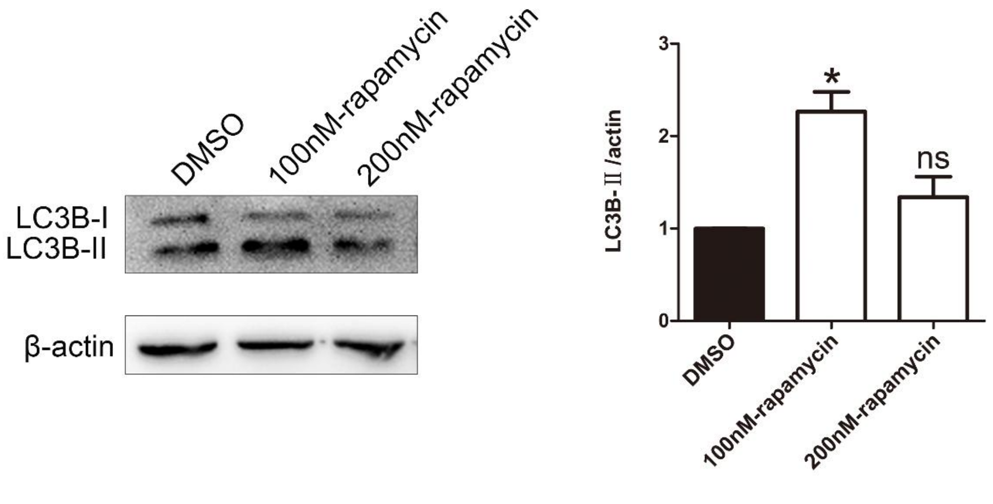

Supplement: Supplementary file 1 — Supplementary figure S1. [file thnov10p11549s1.pdf]
